# Reviewing the Entity: Retropharyngeal Fibrolipoma and a Rare Case Report

**Published:** 2015-11

**Authors:** Behera Ganakalyan, Samal Dilllip Kumar

**Affiliations:** 1 *Department of ENT and Head Neck Surgery, All India Institute of Medical Sciences, Saket nagar, Bhopal, Madhya Pradesh, India.*

**Keywords:** Adipocyte, Fibrolipoma, Obstructive sleep apnoea (OSA), Retro pharyngeal

## Abstract

**Introduction:**

Fibrolipoma, a subtype of lipoma is painless, well-circumscribed, slow-growing, submucosal benign adipocyte tumour. It is uncommon in the oral cavity and oropharyngeal region, with rare incidence in the retropharynx even rarest in pediatric age group.

**Case Report:**

A very unusual case of fibrolipoma is presented in a pediatric patient, who had a huge retropharyngeal fibrolipoma and who presented with breathing difficulty and increasing stridor. It was managed by intro-oral approach excision.

**Conclusion:**

Although rare, retropharyngeal benign tumours should be kept in mind during the differential diagnosis of a paediatric stridor case. Early diagnosis is the key for a better outcome and to alleviate the worsening morbidity.

## Introduction

Lipomas are common benign soft tissue mesenchymal tumours involving tissues containing lipocytes. These are commonly found in middle-aged population with no gender predilection. The oral cavity is an uncommon site of presentation comprising 1-4% of cases only (1). In the oral cavity, common sites are buccal mucosa, floor of mouth, and tongue. Although few cases of simple lipomas of the retropharyngeal region were described in literature, fibrolipomas are a very rare entity. These patients usually present late with complaint of difficulty in swallowing, breathing difficulty, and change in voice. Diagnoses of retropharyngeal pathologies are very important to rule out other neoplastic lesions, which can be either primary or metastatic and infective pathologies. Complete surgical excision is the treatment of choice, when indicated. We had reviewed the PubMed database for the term “retropharyngeal lipoma/ retropharyngeal fibrolipoma” and only found 14 reported cases in the last 14 years (from 2000 to 2013). All case reports were summarized for age and sex distribution, mode of presentation, and treatment modality (2-15) (Table. 1). 

Simple lipoma was the most common variant among them. Here, a rare case of huge retropharyngeal fibrolipoma is reported. Most likely, this is the first reported case in a very young patient (2years of age), who showed symptoms of obstructive sleep apnea and stridor.

**Table1:** Showing Review of literatures (Retropharyngeal lipoma) from 2000-2013

**Sl no.**	**Author**	**Patient information**	**Presentations**	**Management**	**Published year**
1	Akhtar J et al(2)	76 yr/ Male	Dysphagia, Hoarseness	External cervical approach	2001
2	Hockstein NG et al(3)	64 yr/ Male	Obstructive sleep apnoea	Followed up with serial MRI	2002
3	Shivakumar AM et al(4)	12 yr/ male	Nasal obstruction/ Dysphagia/ snoring	Transoral excision	2004
4	Namyslowski G et al(5)	40yr/ Male	Obstructive sleep apnoea	Transcervical excision	2006
5	Behnoud F. MD et al(6)	60 yr/ Female	Dysphagia, Snoring	Transcervical excision	2006
6	Gong W et al(7)	11 yr/ Female	Obstructive sleep apnoea	Surgical excision	2006
7	Piccin O et al(8)	73 yr / female	Obstructive sleep apnoea	Transoral excision	2007
8	Radhakrishna Pillai OS et al(9)	42 yrs/ Male	Dyspnoea	Intra-oral excision	2007
9	Gupta P et al(10)	65 yr/ Male	Dysphagia, dysphonea and dyspnoea	Transcervical excision	2007
10	Lakadamyali H et al(11)	75 yr/ Male	Dyspnoea, Dysphagia and OSA	Follwed up	2008
11	Huang, H.-C.et al(12)	17 yr/ Male	OSA and dysphagia	Transoral excision	2008
12	Bohm KC et al(13)	15 yr/ Female	Asymptomatic parapharyngeal mass	Transoral excision	2011
13	Lee HK et al(14)	69 yr/ Female	Dysphagia and dyspnoea	Transcervical excision	2013
14	Chua DY etal(15)	71 yr/ Male	Mild dysphagia	Transoral excision	2013

## Case Report

A 2-year-old male baby was referred to our otolaryngology and head & neck surgery outpatient department with a progressively worsening difficulty in swallowing and noisy breathing for the last 3 months. The patient had no history of any fever, recurrent cough or cold, vomiting, seizure, or nasal regurgitation of food particles. He had no history of any foreign body ingestion or any contact history of tuberculosis. He was examined clinically and found to have a smooth mucosa covering a retropharyngeal bulge, which was compromising the oropharyngeal inlet and abutting both tonsils. Palatal movements and bilateral tonsils were normal. The patient showed symptoms of stridor and oxygen saturation was near 84% in room air. The patient was admitted in the paediatric intensive care unit and kept under close watch to monitor any vital deterioration. 

**Fig1:**
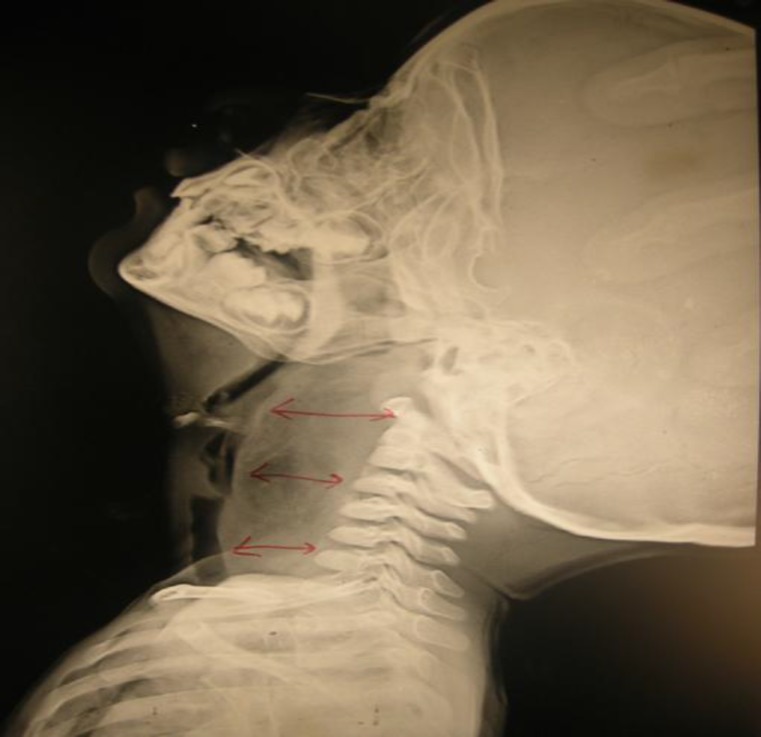
X Ray soft tissue neck (Lateral view) showing increased prevertebral soft tissue shadow from C1 to C7 vertebra displacing the airway anteriorly without any air-fluid level

Fibre-optic laryngoscopy was performed with precaution and results were suggestive of a large retropharyngeal bulge with pooling of saliva in both pyriform sinuses. The endolarynx could not be examined properly. The patient underwent radiological evaluation with X-ray soft tissue neck (lateral view), which showed increased prevertebral soft tissue shadow with predominant fat lucency from C1 to C7 vertebrae, displacing the larynx and trachea anteriorly without any significant airway narrowing. No calcification or air fluid level was observed (Fig.1). 

**Fig 2:**
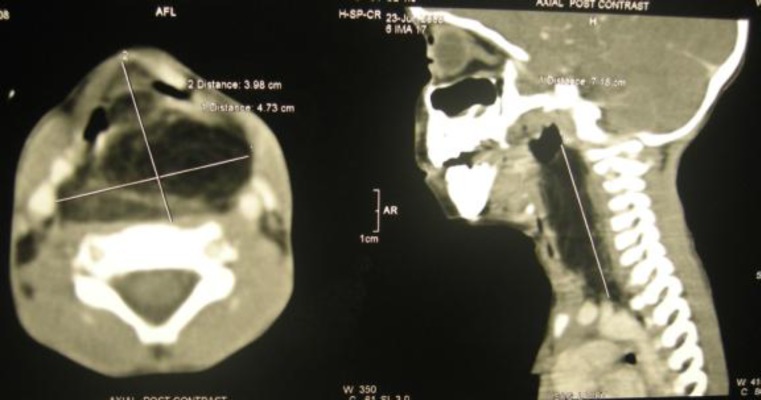
Reconstructed Contrast enhanced Computed tomography (Axial and Sagittal section) showing a fat attenuating mass of 3.98X4.7X7.0 cm in the retropharynx from C1 to D2, displacing the larynx anteriorly and bilateral and displacing the carotid laterally

Contrast enhanced computed tomography from the skull base to the diaphragm showed a 3.98 x 4.7 x 7.0 cm well-defined, fat attenuating mass with enhancing septa within, extending from C1 to D2 level in the retropharyngeal region without any calcification or cystic spaces. The mass was displacing the airway anteriorly and bilaterally and displacing the carotids laterally. Fat planes with prevertebral muscles and underlying vertebrae were normal (Fig. 2). A provisional diagnosis of retropharyngeal lipoma or teratoma was made. Intraoral surgical excision was planned.

**Fig 3:**
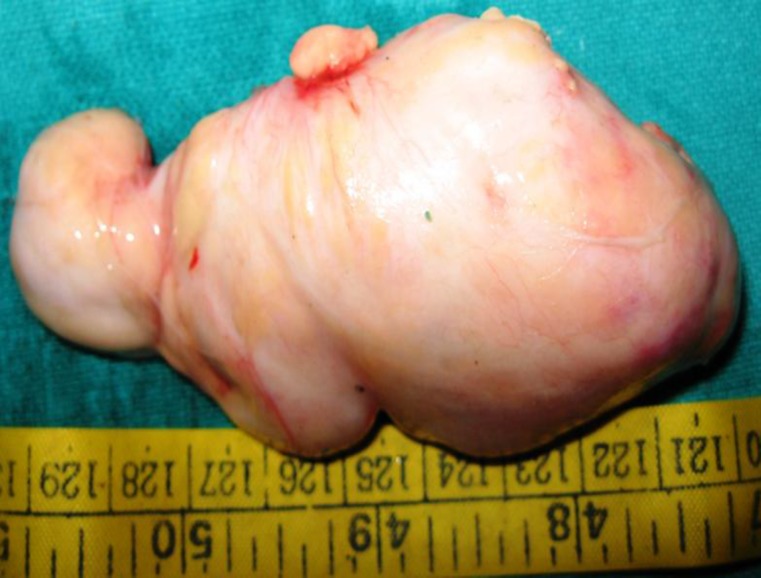
Smooth encapsulated excised retropharyn- geal tumor (8.0x5.0cm

**Fig 4:**
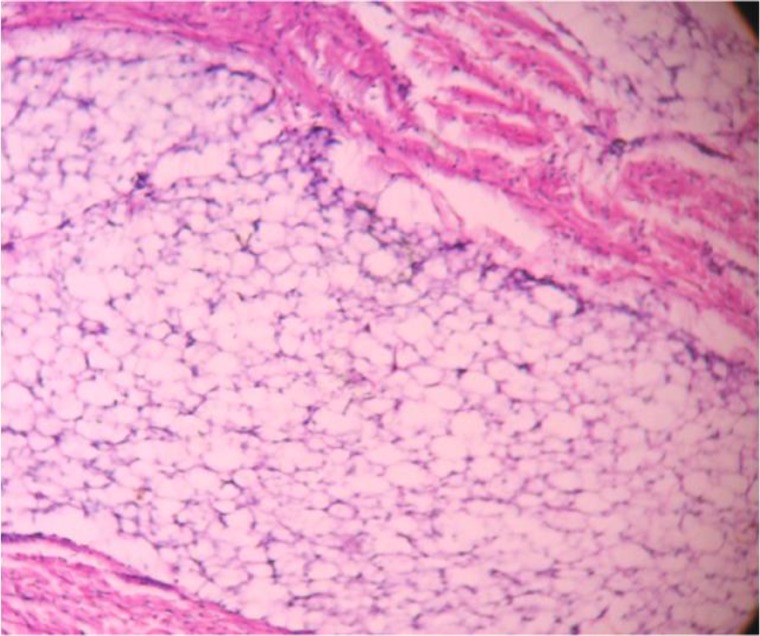
Histopathology: Haematoxylin & Eosin stain (100X) in low power showing adipocytes and fibrous septa

Under general anesthesia, direct laryngoscopy guided oral intubation was performed. A longitudinal mucosal incision was made in the retropharynx. A well-circumscribed, capsulated, firm tumor was delineated and separated from surrounding tissue by blunt dissection and was delivered in toto intra-orally. Primary wound closure was done after achieving haemostasis. The excised specimen was sent for histopathological examination after fixing with formalin. Intra-operative and immediate post-operative periods were uneventful and the patient was kept on Ryle’s tube feeding post-operatively for 2 weeks. Histopathological examination showed a pale, firm, smooth, capsulated soft tissue tumor of a 8.0 X 5.0 cm size (Fig. 3). Microscopic examination showed round to oval adipocytes admixed with collagen and fibrous septa (Fig. 4), which was suggestive of fibrolipoma. The patient was followed up at 3 months, and then had 6 monthly follow-ups for 2 years. No recurrence was observed.

## Discussion

Lipomas are the most common mature adipocyte soft tissue tumours, mostly affecting the trunk, shoulder, neck, and axilla (16) with a distribution of 13% in the head and neck region (17). Involvement of the oral cavity is rare and comprises 4.4% of all soft tissue tumours (18). In the oral cavity, the buccal mucosa is the most common site involved; followed by the tongue, palate, floor of mouth, and vestibule (18). Retropharynx is still an uncommon site of involvement and especially for the fibrolipoma variant. It has an equal gender distribution with a mean age of presentation at the 6th decade (19). Although exact aetiology is not clear, heredity, fatty degeneration, hormonal changes, trauma, infections, infarction, and chronic irritations are possible contributing factors described in literature. Lipomas can be classified histopathologically into simple lipoma, fibrolipoma, spindle cell lipoma, intramuscular or infiltrating lipoma, salivary gland lipoma, and myxoid lipoma (20). Among them fibrolipomas are a variant of lipoma with histopathological features characterized by adipocytes admixed with significant fibrous components (21).

The retropharyngeal space is a potential space with bucopharyngeal fascia anteriorly and alar fascia posteriorly, separating it from the dangear space. It extends from the skull base to T4 vertebra caudally. It contains fatty tissue and lymph nodes and serves as a potential space for spread of infections and malignancy. Retropharyngeal lipomas usually present in later stages with progressive difficulty in swallowing, breathing difficulty, and change in voice. Diagnosis is made by clinical and radiological investigations and the final diagnosis depends upon the histopatho- logical examination after complete surgical excision. Furthermore, fibrolipomas have a greater proliferative activity in comparison to other variants of lipomas, thus accurate diagnosis is essential (1). Retropharyngeal tumours should be differentiated from primary lymphoma of the retropharynx, metastatic lymphadenopathy, and retropharyngeal abscess by proper systemic examinations and radiology. Prior informed and written consent for surgical tracheostomy should be taken, due to the anticipated difficulty of endotracheal intubation. Surgical excision either intra-orally or by transcervical route is the primary modality of treatment; but can be followed up in frail patients with poor general medical conditions, with serial radiological investigations. Fibrolipomas have an excellent prognosis with very rare recurrence on prolonged follow up. Although malignant transformations are unlikely, progression to liposarcoma has been reported in literature (20). 

## Conclusion

Fibrolipomas are well-circumscribed, slow growing soft tissue adipocyte tumours. Clinically these are asymptomatic unless they increase in size. Retropharyngeal fibrolipomas should be differentiated from other neoplastic or infective pathologies. Complete surgical excision is the choice treatment modality. A well-experienced surgeon needs a well-equipped setup and trained anesthetists for better airway maintenance during intraoral excision.

## References

[1] Fregnani ER, Pires FR, Falzoni R, Lopes MA, Vargas PA (2003). Lipomas of the oral cavity: clinical findings, histological classification and proliferative activity of 46 cases. Int J Oral Maxillofac Surg.

[2] Akhtar J, Shaykhon M, Crocker J, D'Souza AR (2001). Retropharyngeal lipoma causing dysphagia. Eur Arch Otorhinolaryngol.

[3] Hockstein NG, Anderson TA, Moonis G, Gustafson KS, Mirza N (2002). Retropharyngeal lipoma causing obstructive sleep apnoea: case report including five-year follow-up. Laryngoscope.

[4] Shivakumar AM, Naik AS, Shetty DK, Yogesh BS (2004). Lipoma of the retropharyngeal space. Indian J Pediatr.

[5] Namyslowski G, Scierski W, Misiolek M, Urbaniec N, Lange D (2006). Huge retropharyngeal lipoma causing obstructive sleep apnoea: A case report. Eur Arch Otorhinolaryngol.

[6] Behnoud FA, Hashemian F (2006). A case report of huge retropharyngeal lipoma. Iran J Otorhinolaryngol.

[7] Gong W, Wang E, Zhang B, Da J (2006). A retropharyngeal lipoma causing obstructive sleep apnoea in a child. J Clin Sleep Med.

[8] Piccin O, Sorrenti G (2007). Adult obstructive sleep anoea related to nasopharyngeal obstruction: a case of retropharyngeal lipoma and pathogenetic considerations. Sleep Breath.

[9] Radhakrishna Pillai OS, Vijayalakshmi S, Adarsha TV, Thahir M, Gopinathan UK, Mohammed N (2007). Retropharyngeal lipoma-a case report. Indian J Otolaryngol Head Neck Surg.

[10] Gupta P, Deo RP, Udupa KV, Ravi HR, Pai SA (2008). A case of retropharyngeal Lipoma. Indian J Surg.

[11] Lakadamyali H, Ergun T, Lakadamyali H, Avci S (2008). A giant retropharyngeal lipoma showing no change in clinical prrsentation and size within a two-year follow-up: a case report. Kulak Burun Bogaz Ihtis Derg.

[12] Huang HC, Li HY (2009). Retropharyngeal fibrolipoma: A counterchanging obstructive pattern in sleep apnea. Int J Pediatr Otorhinolaryngol.

[13] Bohm KC, Birman MV, Silva SR, Lesperance MM, Marentette LJ, Beyer GR (2011). Ossifying lipoma of c1-c2 in an adolescent. J Pediatr Orthop.

[14] Lee HK, Hwang SB, Chung GH, Hong KH, Jang KY (2013). Retropharyngeal spindle cell/ pleomorphic lipoma. Korean J Radiol.

[15] Chua DY, Lim MY, Teo DT, Hwang SY (2013). Retropharyngeal lipoma with parapharyngeal extension: is transoral excision possible. Singapore Med J.

[16] Said-Al-Naief N, Zahurullah FR, Sciubba JJ (2001). Oral spindle cell lipoma. Ann Diagn Pathol.

[17] Castilho RM, Squarize CH, Nunes FD, Pinto Júnior DS (2004). Osteolipoma: a rare lesion in the oral cavity. Br J Oral Maxillofac Surg.

[18] Furlong MA, Fanburg-Smith JC, Childers EL (2004). Lipoma of the oral and maxillofacial region: Site and subclassification of 125 cases. Oral Surg Oral Med Oral Pathol Oral Radiol Endod.

[19] Manor E, Sion-Vardy N, Joshua BZ, Bodner L (2011). Oral lipoma: analysis of 58 new cases and review of the literature. Ann Diagn Pathol.

[20] Studart-Soares EC, Costa FW, Sousa FB, Alves AP, Osterne RL (2010 ). Oral lipomas in a Brazilian population: a 10-year study and analysis of 450 cases reported in the literature. Med Oral Patol Oral Cir Bucal.

[21] Khubchandani M, Thosar NR Bahadure RN, Baliga MS, Gaikwad RN (2012 ). Fibrolipoma of buccal mucosa. Contemp Clin Dent.

